# Oral Administration of Herbal Mixture Extract Inhibits 2,4-Dinitrochlorobenzene-Induced Atopic Dermatitis in BALB/c Mice

**DOI:** 10.1155/2014/319438

**Published:** 2014-07-16

**Authors:** Soon Re Kim, Han-Seok Choi, Hye Sook Seo, Jin Mo Ku, Se Hyang Hong, Hye Hyun Yoo, Yong Cheol Shin, Seong-Gyu Ko

**Affiliations:** ^1^Laboratory of Clinical Biology and Pharmacogenomics, Department of Preventive Medicine, College of Oriental Medicine, Kyung Hee University, Seoul 130–701, Republic of Korea; ^2^Department of Pharmacy, College of Pharmacy, Hanyang University, Ansan, Gyeonggi-do 426–791, Republic of Korea

## Abstract

CP001 is four traditional herbal medicine mixtures with anti-inflammatory properties. In this study, we investigated the effect of oral administration of CP001 ethanol extract on the 2,4-dinitrochlorobenzene- (DNCB-) induced AD mouse models. For that purpose, we observed the effects of oral administration of CP001 on skin inflammatory cell infiltration, skin mast cells, production of serum IgE, and expression of Th2 cytokine mRNA in the AD skin lesions of DNCB treated BALB/c mice. Histological analyses demonstrated that CP001 decreased dermis and epidermis thickening as well as dermal infiltration induced by inflammatory cells. In addition, CP001 decreased mast cell infiltration in count as well as dermal infiltration induced by inflammatory cells. In the skin lesions, mRNA expression of interleukin- (IL-) 4 and IL-13 was inhibited by CP001. CP001 also reduced the production of IgE level in mouse plasma. In addition, we investigated the effect of CP001 on the inflammatory allergic reaction using human mast cells (HMC-1). In HMC-1, cytokine production and mRNA levels of IL-4, IL-13, IL-6, and IL-8 were suppressed by CP001. Taken together, our results showed that oral administration of CP001 exerts beneficial effects in AD symptoms, suggesting that CP001 might be a useful candidate for the treatment of AD.

## 1. Introduction

Atopic dermatitis (AD) is a most common chronic inflammatory skin disease, affecting about 10 million people in the world, leading to a significant reduction in quality of life, and its incidence is continuously increasing in westernized countries [1, 2]. The pathogenesis of AD is unknown, but the disease seems to be correlated with specific immune and inflammatory mechanisms. The general characteristics of AD include excessive infiltration of inflammatory cells such as lymphocytes, macrophages, and granulated mast cells into the skin lesions, eosinophilia in peripheral blood, and a high level of serum immunoglobulin E (IgE) [3].

Mast cells are tissue-based inflammatory cells of bone marrow origin, which respond to signals of innate and adaptive immunity. They play a major role in immediate hypersensitivity reaction and are activated through the high-affinity IgE receptor, Fc*ε*R [4]. In addition, it has been reported that a large number of mast cells can be found in AD skin lesion. The majority of AD patients have elevated blood IgE level and it is mediated of mast cell activation [5]. Mast cell activation by IgE is release inflammatory mediators, such as histamine, as well as cytokines, including Th2 cytokines, such as IL-4, IL-5, and IL-13. Thus, cell-bound IgE is cross-linked allergens and it is contributed to the development of AD through mediating activation of mast cells localization [6].

Proinflammatory cytokines that are released by activated mast cells, including IL-6 and IL-8, play an important role in allergic inflammation [7]; IL-6 mediates allergic inflammation [8], while IL-8 induces the migration of neutrophils into inflammatory regions as a potent chemotactic cytokine [9, 10].

CP001 is a mixture of four oriental herbal medicines composed of* Houttuynia cordata *Thunb,* Rehmannia glutinosa *Libosch, bark of* Betula platyphylla* var.* japonica,* and* Rubus coreanus *Miq.* Houttuynia cordata *Thunb has long been used in traditional oriental medicine for the treatment of inflammatory diseases. Also, several studies demonstrated that* Houttuynia cordata *Thunb has been associated with a broad range of pharmacological activities, including anti-inflammatory [1], antiviral [11], and anticancer effects [12].* Rehmannia glutinosa *Libosch has traditionally been used as an ingredient herb in East Asian medicine for the effects of hemostasis, activation of blood circulation, and improvement of kidney function [13]. Several studies indicated that* Rehmannia glutinosa* Libosch has antiallergic effects [14] and anti-inflammatory function [15–17].* Betula platyphylla* var.* japonica* is known to have antioxidant, anti-inflammatory, and anticancer effects [18] and inhibits the development of AD in NC/Nga mice [19, 20].* Rubus coreanus *Miq. is a type of red raspberry that grows wild in Korea, Japan, and China. The fruit, known as “*Bokbunja*” in Korean, has been used in traditional oriental medicine for reducing the risk of diseases such as asthma and allergy. It is also known that* Rubus coreanus *Miq. has anti-inflammatory and antioxidative activities [21–23]. These collective observations indicate that CP001 may be good candidate for control of AD and beneficial in the treatment of human allergic disorders. Therefore, in our previous study, we already confirmed that topical application of KM110329 (CP001 modifying herbal mixture) inhibits the atopic dermatitis in ovalbumin- and DNCB-induced mouse model.

Therefore, in this study, we investigated whether 30% ethanol extract of CP001 oral administration has anti-inflammatory activity in 2,4-dinitrochlorobenzene- (DNCB-) induced AD mice model. In addition, we also investigated whether 30% ethanol extract of CP001 has antiallergic effect inhibiting cytokine production in human mast cells, HMC-1.

## 2. Material and Method

### 2.1. Preparation of CP001

CP001 was prepared by Hanpoong Pharmaceutical (Jeon-ju, Korea) following good manufacturing practices (GMP) procedure. CP001 is 30% ethanol extracted brown-colored powder, and it is composed of* Houttuynia cordata *Thunb,* Rehmannia glutinosa *Libosch, bark of* Betula platyphylla* var.* japonica*, and* Rubus coreanus *Miq. The powder from the extract was dissolved in distilled water for* in vivo* and* in vitro* experiments.

### 2.2. Animals

Six-week-old male BALB/c mice were purchased from Orient (Sung-nam, Korea). The mice were randomized into 6 groups (normal, DNCB, and 25, 50, 100, and 200 mg/kg (CP001)), each comprising five mice. All mice were kept under pathogen-free environment and allowed free access to the diet and water. All procedures performed on the mice were approved by the animal care center of Kyung-Hee University (Approval Number KHUASP (SE)-2012-004).

### 2.3. Induction of AD-Like Skin Lesions and CP001 Treatment

**Figure 1:**
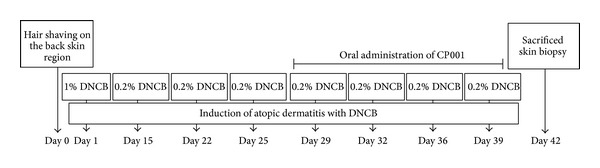
Protocols for induction of atopic dermatitis in mouse model. Shaved dorsal regions of the mice were sensitized with DNCB solution. Male BALB/c mice were epicutaneously sensitized with 200 *μ*l of a 1% DNCB solution on day 1. Two weeks later, dermatitis was induced with 200 *μ*l of 0.2% DNCB solution at the intervals shown in the figure. CP001 was orally administrated from the 3rd week during sensitization together with DNCB.

Induction of AD-like skin lesions procedure is described in Figure 1. For that purpose, mice back skin was painted dermally with 200 *μ*L of a 1% DNCB using 1 × 1 cm patches after shaving. Two weeks after sensitization, the back skin was challenged with 200 *μ*L of a 0.2% DNCB solution twice per week. This procedure was repeated for 2 weeks and CP001 was orally administrated together. At the end of the experiment, mice were sacrificed by CO_2_ inhalation, and samples were collected.

### 2.4. Histological Analysis

A portion of the skin biopsies were fixed in 4% paraformaldehyde (PFA) and embedded in Tissue-Tek optical cutting temperature (O.C.T.) compound (Tissue-Tek, Sakura, AA Zoeterwoude, the Netherlands) on dry ice. Skin sections of 20 *μ*m were cut and stained with hematoxylin and eosin (H & E) for inflammatory cells or with toluidine blue for mast cells counts and examined under light microscopy (Olympus). Mast cells were counted in 10 parts of high-power fields (HPF) at 400x magnification.

### 2.5. Enzyme-Linked Immune Sorbent Assay

After final CP001 administration, whole blood samples were collected by cardiac puncture for measurement of blood IgE level. The blood was placed in Vacutainer tubes containing EDTA (BD science, NJ, USA) and blood plasma was isolated. Total IgE levels in plasma were determined by sandwich ELISA using the BD PharMingen mouse IgE ELISA set. Briefly, plates were coated with capture antibody in ELISA coating buffer (Sigma-Aldrich) and incubated overnight at 4°C. Plates were washed with PBS-Tween 20 (0.05%) and subsequently blocked (10% FBS in PBS) for 1 h at 20°C. Serial dilutions of standard antigen or sample in dilution buffer (10% FBS in PBS) were added to the plates and plates were incubated for 2 h at 20°C. After washing, biotin-conjugated anti-mouse IgE and SAv-HRP (streptavidin-horseradish peroxidase conjugate) were added to the plates and plates were incubated for 1 h at 20°C. Finally, tetramethylbenzidine (TMB) substrate solution was added to the plates and after 15 min incubation in the dark, a 2 N H_2_SO_4_ solution was added to stop the reaction. Optical densities were measured at 450 nm on an automated ELISA reader (Versa Max, Molecular Devices, CA, USA). IL-6 and IL-8 levels were measured in HMC-1 supernatant by sandwich ELISA using BD Pharmingen human ELISA set. The sandwich ELISA procedures were performed by following the same protocols described above.

### 2.6. Cytokine Analysis by Real-Time PCR

Mice skin was immediately frozen in liquid nitrogen and kept at −70°C until use. For real-time PCR assay, mice skin was homogenized with Ultra-Turrax T10 (IKA labortechnik, Seoul, Korea) and RNA extraction was performed using TRIzol (Invitrogen life technologies, NY, USA). RNA content was measured using the NanoDrop ND-1000 spectrophotometer (NanoDrop Technologies Inc.). 1 g of total cellular RNA from each sample was reverse transcribed using cDNA synthesis kit (TaKaRa, Japan). Quantitative PCR was performed using SYBR green iMaster and a LightCycler 480 (Roche, Switzerland).

### 2.7. Reverse Transcription-Polymerase Chain Reaction (RT-PCR)

Cells were harvested by centrifugation and the pellet was washed with ice-cold PBS. RNA was isolated from the pellet using easy-blue RNA extraction kit (iNtRON Biotech, Republic of Korea) according to the manufacturer's instructions. Isolated RNA content was measured using the NanoDrop ND-1000 spectrophotometer (NanoDrop Technologies Inc.). 2 *μ*g of total cellular RNA from each sample was reverse transcribed using cDNA synthesis kit (TaKaRa, Japan). PCR was conducted out in a 20 *μ*L reaction mixture consisting of DNA template, 10 pM of each gene-specific primer, 10x Taq buffer, 2.5 mM dNTP mixture, and 1 unit of Taq DNA polymerase (Takara, Japan). PCR was performed using the specific primer and primer sequences for human IL-6, IL-8, and GAPDH are shown in Table 1.

### 2.8. HPLC Analysis

Ellagic acid, quercitrin hydrate, and catalpol were purchased from Sigma Chemicals (Saint Louis, MO). Purity of standard compounds was guaranteed to be higher than 95% by HPLC. HPLC grade acetonitrile, methanol, and formic acid were purchased from J. T. Baker (Phillipsburg, NJ). Catalpol, ellagic acid, and quercitrin were chosen as Marker compounds to standardize the extract sample. CP001 was dissolved in distilled water at a concentration of 100 mg/mL and the solution was filtered through a 0.45 *μ*m membrane filter. A 10 *μ*L aliquot of the sample solution was injected into a HPLC system (Agilent Technologies, Palo Alto, CA). The sample was analyzed on a Capcell Pak UG120 C_18_ analytical column (250 × 4.6 mm, 5 *μ*m; Shiseido, Japan).

### 2.9. Statistical Analysis

Statistical analyses presented as the mean ± standard error of the mean (SEM) and were analyzed for statistical significance using the unpaired Student's* t*-test.* P* value < 0.05 was considered statistically significant.

## 3. Results

### 3.1. Oral Administration of CP001 Decreases Infiltration of Inflammatory Cells into AD-Like Skin Lesions

**Figure 2:**
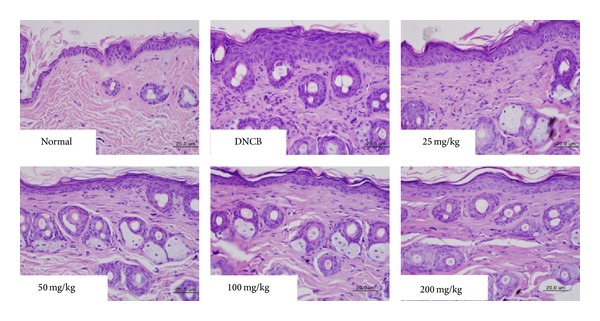
Histological features of AD-like skin lesions treated with CP001. The skin sections were stained with hematoxylin and eosin. Inflammatory cells infiltration into the dermis was measured after treatment with CP001 in the presence of DNCB. Sections were evaluated using microscope at an original magnification of 400x.

**Figure 3:**
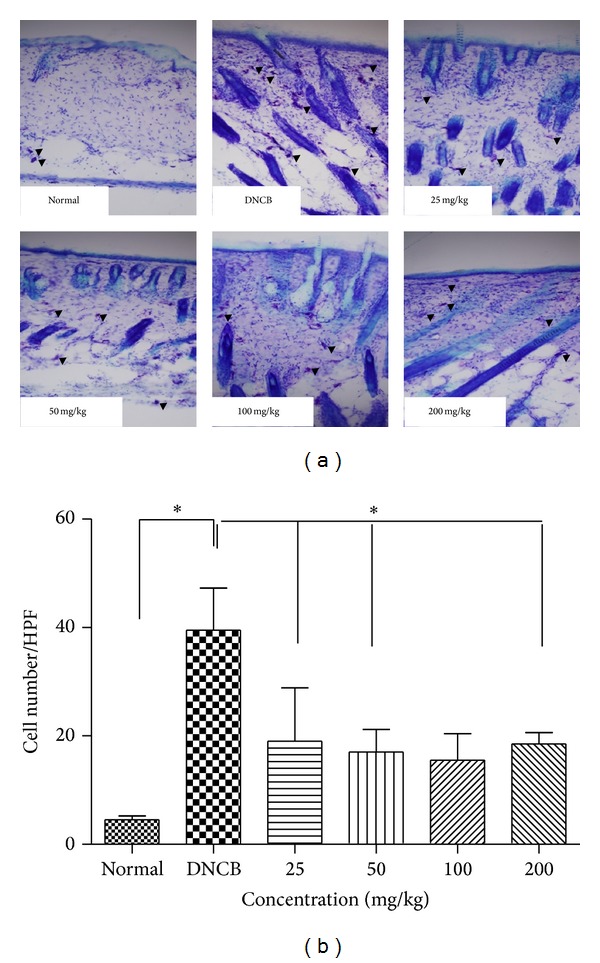
The measurement of mast cells number in AD-like skin lesions treated with CP001. The skin sections were stained with toluidine blue for mast cells staining. Sections were evaluated using microscope at an original magnification of 400x. The data are presented as mean ± SD from five animals in each group. *P < 0.05.

To determine whether CP001 decreases infiltration of inflammatory cells into AD-like skin lesions, we performed H & E staining on the skin after oral administration of CP001. We observed infiltration of inflammatory cells into the epidermis and dermis in DNCB group, whereas CP001 decreased such infiltration of inflammatory cells into the skin (Figure 2). Moreover, CP001 (25–200 mg/kg) abrogated skin thickening induced by DNCB (Figure 2). Next, we also performed toluidine blue staining for mast cell observation. Repeated cutaneous application of DNCB increased dermal mast cell number. However, this feature was significantly suppressed by CP001 (Figure 3).

### 3.2. CP001 Administration Downregulates mRNA Expression of Th2 Cytokines

**Figure 4:**
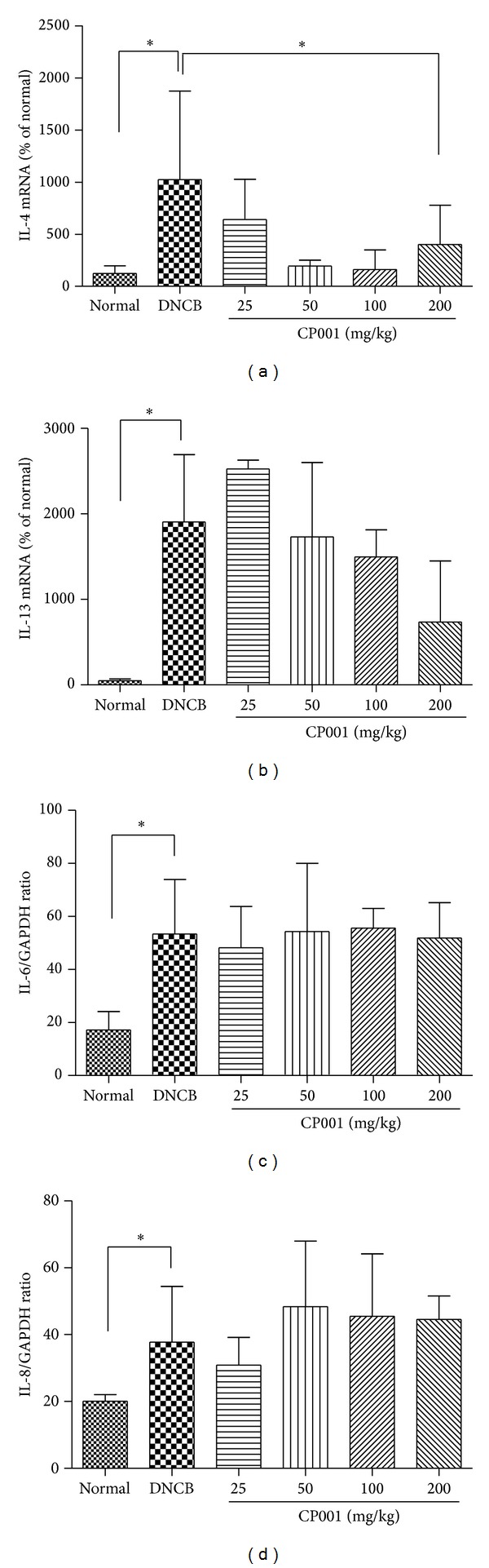
Effect of CP001 on the cytokine mRNA expression in mouse skin tissue. The IL-4, IL-13, IL6, and IL-8 mRNA expression were measured by real-time PCR (a), (b) and RT-PCR (c), (d) in mouse skin tissue. The columns and the error bars represent mean ± SD (*n* = 5 mice/group). *P < 0.05.

The Th2 type cytokines are important in an acute phase of AD whereas mixed Th2/Th1 type inflammation is characteristic to a chronic phase of AD. To determine whether CP001 decreases Th2 type cytokines expression, we performed real-time PCR to measure IL-4 and IL-13 levels. We found that oral administration of CP001 decreased IL-4 mRNA expression in AD-like skin lesions (Figure 4(a)). We also found that CP001 administration decreased IL-13 mRNA expression in AD-like skin lesions in a dose-dependent manner (Figure 4(b)). In histology analysis, repeated cutaneous application of DNCB increased dermal mast cell number and this feature was suppressed by CP001 oral administration. Activated mast cells secrete various chemokines and cytokines including IL-6 and IL-8. To determine whether CP001 decreases IL-6 and IL-8 cytokines mRNA levels, we performed RT-PCR analysis in AD-like skin lesions. Oral administration of CP001 did not affect the suppression of IL-6 and IL-8 mRNA expression in AD-like skin lesions (Figures 4(c) and 4(d)).

### 3.3. CP001 Administration Downregulates Serum IgE Concentration

**Figure 5:**
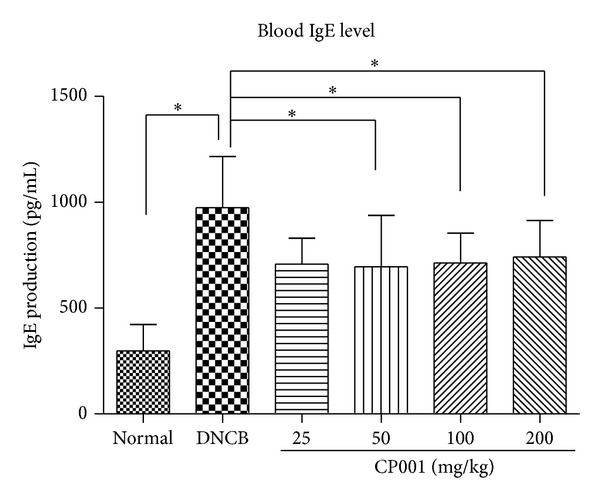
Measurement of plasma IgE level. Total IgE level was determined by ELISA. The columns and the error bars represent mean ± SEMs (*n* = 4 mice/group). *P < 0.05.

Hyperproduction of IgE is a major characteristic of AD and patients with AD often exhibit elevated levels of total and allergen specific IgE antibodies (Abs) in their serum. To further test whether suppression of the progression of AD-like skin lesions by CP001 is associated with serum IgE levels, we performed total IgE ELISA assay. We found that total IgE levels were dramatically elevated in DNCB-treated group compared with normal group. However, increased serum IgE levels induced by DNCB were significantly decreased by CP001 treatment (Figure 5).

### 3.4. Effect of CP001 on PMA Plus A23187-Induced IL-6 and IL-8 Expression in HMC-1

**Figure 6:**
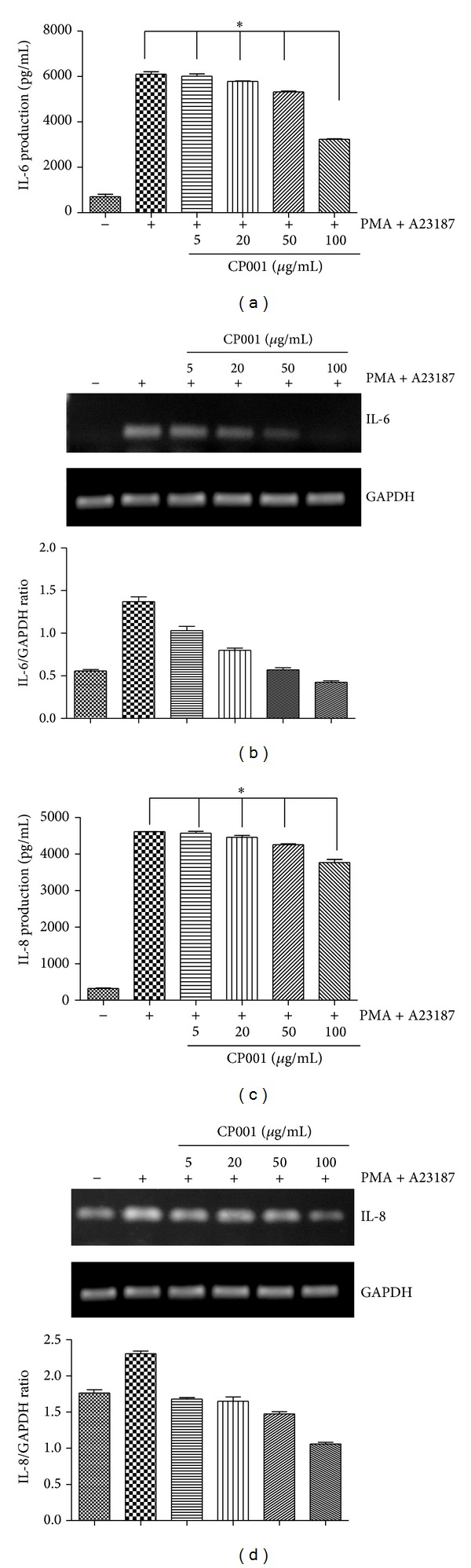
Effect of CP001 on PMA plus A23187-stimulated proinflammatory cytokine expression in HMC-1. HMC-1 were pretreated with various concentrations of CP001 for 1 h and then treated with PMA and A23187 for 24 h. The levels of IL-6 and IL-8 in culture supernatants were measured by ELISA assay (a), (c). The IL-6 and IL-8 mRNA levels were measured by RT-PCR (b), (d). Data represent the mean ± SEMs of three independent experiments.

Next, we investigated whether CP001 affects production of IL-6 and IL-8 in HMC-1. For that purpose, mast cells were pretreated with various concentrations of CP001 for 1 h and then treated with PMA and A23187 for 24 h. The levels of IL-6 and IL-8 in culture supernatants were measured by ELISA assay. We found that IL-6 secretion induced by PMA and A23187 was significantly suppressed by CP001 (Figures 6(a) and 6(c)). We also performed RT-PCR to measure IL-6 and IL-8 mRNA expression in HMC-1. We observed that IL-6 and IL-8 mRNA induced by PMA and A23187 were decreased by CP001 (Figures 6(b) and 6(d)).

### 3.5. Effect of CP001 on PMA Plus A23187-Induced Th2 Cytokine Expression in HMC-1

**Figure 7:**
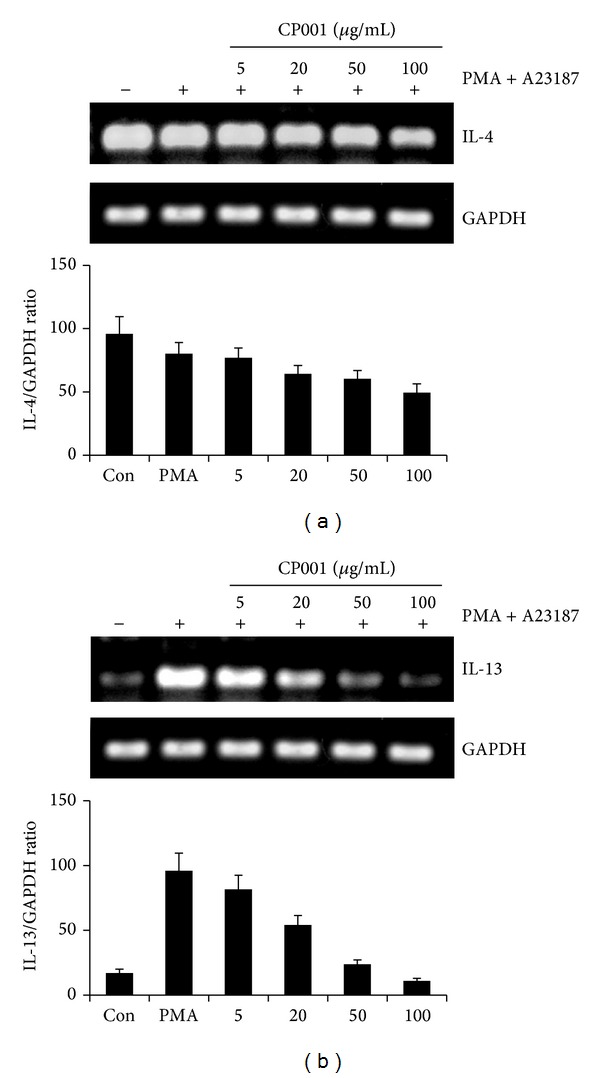
Effect of CP001 on PMA plus A23187-stimulated Th2 cytokine expression in HMC-1. HMC-1 were pretreated with various concentrations of CP001 for 1 h and then treated with PMA and A23187 for 24 h. The IL-4 and IL-13 mRNA levels were measured by RT-PCR (a), (b). Data represent the mean ± SEMs of three independent experiments.

CP001 administration decreased IL-4 and IL-13 mRNA expression in AD-like skin lesions (Figure 7(b)). Therefore, we characterized the regulatory effect of CP001 on IL-4 and IL-13 mRNA expression in HMC-1 using RT-PCR. We found that IL-13 expression induced by PMA and A23187 was significantly suppressed by CP001 (Figure 7(a)). IL-4 expression level was not increased by PMA and A23187, but it is suppressed by CP001 (Figure 7(a)).

### 3.6. HPLC Analysis

**Figure 8:**
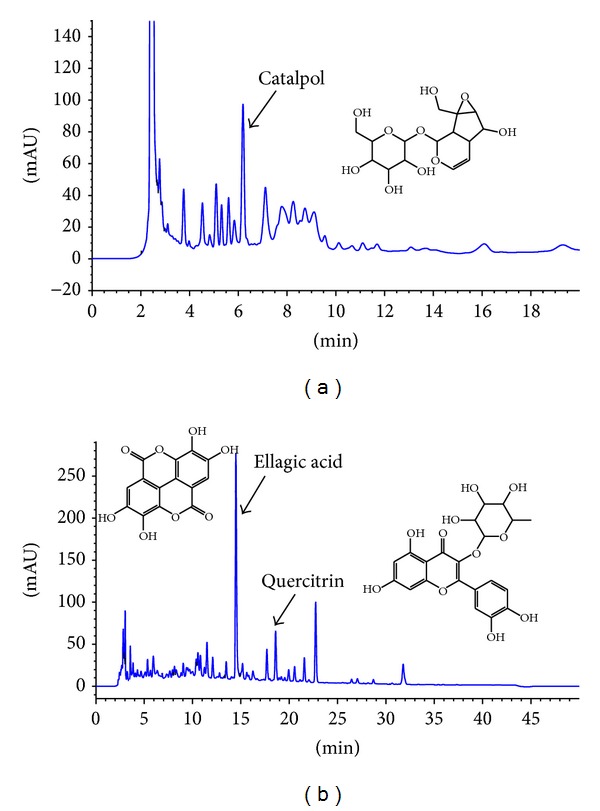
Typical HPLC chromatograms of CP001 for (a) catalpol, (b) ellagic acid, and quercitrin.

To further evaluate the effective compounds of CP001 extract, HPLC analysis was employed. In order to analyze catalpol, the mobile phase consisted of water (W) and methanol (M) and the flow rate was 1 mL/min. The gradient elution program was used as follows. The initial composition of the mobile phase was 97 : 3 (W : M), which was linearly changed to 95 : 5 (W : M) over 1 min and changed to 91 : 9 (W : M) for 9 min. At 11 min, the composition of mobile phase returned to the initial condition, which was maintained for 9 min for column reequilibration. Chromatograms were acquired at 210 nm by UV detection (Figure 8). For ellagic acid and quercitrin, the mobile phase consisted of 0.1% formic acid (F) and acetonitrile (A) and the flow rate was 1 mL/min. The gradient elution program was used as follows. The initial composition of the mobile phase was 90 : 10 (F : A), which was linearly changed to 85 : 15 (F : A) over 5 min and changed to 60 : 40 (F : A) for 35 min. At 41 min, the composition of mobile phase returned to the initial condition, which was maintained for 9 min for column reequilibration. Chromatograms were acquired at 254 nm by UV detection. The retention times of catalpol, ellagic acid, and quercitrin were 6.2, 14.4, and 18.6 min, respectively (Figures 7(a) and 7(b)). The concentrations of catalpol, ellagic acid, and quercitrin in the extract sample were determined using HPLC analysis as described above. The extract was standardized to contain 1.8% catalpol, 0.4% ellagic acid, and 0.3% quercitrin.

## 4. Discussion

AD is a chronic inflammatory disease, which is accompanied by erythema, edema, and scaling in AD skin lesions [24]. Recently, Korean medicine has been the subject of increased interest for its potential in the treatment of inflammatory diseases, including atopic dermatitis and airway inflammation [16, 17, 25, 26]. The present study demonstrates that oral administration of Korean herbal mixture extract, CP001, inhibits DNCB-induced AD. We observed that CP001 decreases infiltration of inflammatory cells into AD-like skin lesions and dermal mast cell number.

Generally, steroid therapy is used for AD treatment, but it cannot be administrated over the long term because of the many side effects. Therefore, we find a new drug, which is effective in the treatment of AD without any side effects. Recently, we reported that topical application of KM110329 (CP001 modified drug) reduced ovalbumin- and DNCB-induced atopic dermatitis [23]. Therefore, we were wondering whether CP001 oral administration may inhibit DNCB-induced atopic dermatitis.

Mast cells degranulation can be regulated by the recruitment, trafficking, and function of inflammatory response. For example, IL-4 and IL-13 induce cell adhesion molecules on endothelium which can be recruitment of leukocytes [27–29]. Also, the production of IL-4 cytokine in epidermal cells has been known to be the main factor for initiation of AD [30]. In our data, we show that cytokine production and mRNA levels of IL-4, IL-13, IL-6, and IL-8 were suppressed by CP001 in HMC-1. Also, quantitative real-time PCR of the skin lesions also showed that oral administration of CP001 diminished the mRNA level of IL-4 and IL-13 in the AD-like skin lesions. In addition, we found that CP001 reduces mast cell in DNCB-induced AD-like skin lesion. It seems that inhibition of infiltration of mast cell downregulates secretion of IL-4 and IL-13 cytokines and it may inhibit recruitment of leukocytes. Thus, mast cell may be main factor for suppression of Th2 cytokines in the AD-like skin lesions by oral administration of CP001.

IgE is mediator of mast cell activation and we observed that CP001 oral administration reduced elevated blood IgE levels induced by repeated DNCB sensitization.

CP001 also suppressed IL-6 secretion and elevated IL-6 and IL-8 mRNA expression induced by PMA and A23187 in HMC-1. It seems that the reduction of infiltration of mast cells is related to decrease of degranulation of mast cells and maturation of eosinophils suppressing the release of various inflammatory cytokines.

## 5. Conclusion

Our present study clearly demonstrates that CP001 suppresses the progression of AD induced by DNBC. In addition, inflammatory related cytokine production and mRNA levels of IL-4, IL-13, IL-6, and IL-8 were suppressed by CP001. This suggests that CP001 might be a useful candidate for the treatment of AD.

## Figures and Tables

**Table 1:** 

Primer name	Sequence (5′-3′)
IL-4	Forward: AAGAACACCACAGAGAGTGAGCTC
	Reverse: TTTCAGTGTGGACTTCCACTC
IL-13	Forward: AGCATGGTATGGAGTGTGGACCTG
	Reverse: CAGTTGCTTTGTGTAGCTGAGCAG
IL-6	Forward: AACCTTCCAAAGATGGCTGAA
	Reverse: CAGGAACTGGATCAGGACTTT
IL-8	Forward: TCAGTGCATAAAGACATACTCC
	Reverse: TGGCATCTTCACTGATTCTTG
GAPDH	Forward: GAGGGGCCATCCACAGTCTTC
	Reverse: CATCACCATCTTCCAGGAGCG
